# Rare Case of a Young Male Presented with Abdominal Pain, Solid Colon Tumors, and Eosinophilia, Followed by Tremendous Thromboembolic Complications and Eventually Diagnosed with Idiopathic Hypereosinophilic Syndrome

**DOI:** 10.1155/2022/1424749

**Published:** 2022-05-18

**Authors:** Tomasz Zemleduch, Anna Czapla, Piotr Kimla, Bartosz Kudliński

**Affiliations:** ^1^Department of Anesthetics, Intensive Care and Emergency Medicine, Collegium Medicum, University of Zielona Gora, Zyty 28, 65-046, Zielona Gora, Poland; ^2^Collegium Medicum, University of Zielona Gora, Zyty 28, 65-046, Zielona Gora, Poland

## Abstract

Hypereosinophilic syndrome (HES) is a rare condition characterized by profound peripheral eosinophilia and various organ dysfunction. Diagnostic criteria and classification of this challenging medical entity changed over time. Elevated absolute eosinophil count with extensive tissue infiltration and signs of organ damage of unknown origin is termed idiopathic HES. Hypereosinophilia is a highly hypercoagulable state; thus, a variety of thromboembolic complications may occur. Only a few reports of idiopathic HES patients with different forms of thrombosis are being published. We document a case of a young male presented with persistent abdominal pain with two eosinophilic colon tumors. The patient suffered from phlegmasia cerulea dolens and portal vein thrombosis, followed by pulmonary embolism and overt disseminated intravascular coagulation (DIC). Corticosteroids successfully reduced and controlled eosinophil level while skilled anticoagulation and supportive management overcome DIC-associated complications.

## 1. Introduction

Hypereosinophilic syndrome (HES) is an extremely rare condition characterized by persistent peripheral eosinophilia and end-organ damage. The incidence ranges from 0.04 to 0.17 per 100,000 person-years [1, 2], and medical knowledge is based mainly on case series reports. Eosinophilic infiltration of the heart, central nervous system, skin, and respiratory tract can occur; thus, clinical manifestation is variable [3]. The precise etiology of HES is unknown, and the diagnosis is made by excluding eosinophilia secondary to allergies, medications, parasites, or viruses (e.g., HIV) and malignancies [3, 4]. Historically, this unusual disease was first described by Hardy and Anderson [5], but diagnostic criteria were established in 1975 by Chusid et al. [6], namely, absolute eosinophil count (AEC) >1.5 × 10^3^/*μ*L that lasted for at least 6 months, as well as signs or symptoms of organ injury. Currently, neither the duration nor the AEC criterion endured. Lower eosinophilia with significant tissue infiltration or AEC elevated >1.5 × 10^3^/*μ*L found twice on different occasions should be sufficient for HES diagnosis [7]. Generally, asymptomatic hypereosinophilia should not be recognized as HES. In addition, a broader understanding of molecular pathophysiology and clinical characteristics of HES resulted in the development of the classification of HES subtypes. The HES main categories are: myeloproliferative (M-HES), lymphocytic (L-HES), familial, overlapping (organ-restricted eosinophilia), associated (present medical condition generating eosinophilia, for example, mastocytosis, inflammatory bowel disease, HIV infection and others) and idiopathic HES [3]. The WHO endorses a different approach to the categorization of eosinophilic syndromes, but with a common idiopathic HES entity as an exclusion diagnosis [4]. Some of the M-HES patients lack defined myeloid neoplasm, but still present myeloproliferative disease-associated signs, such as hepatosplenomegaly, anemia, and thrombocytopenia. Quite often, these patients are found to have a FIP1L1-PDGFRA fusion gene producing a highly active tyrosine kinase, which results in stem cell transformation and peripheral eosinophilia [8]. This mutation warrants imatinib, a tyrosine kinase inhibitor, to be an effective treatment option for this population [9]. In L-HES cases, eosinophilia is a consequence of abnormal production of hematopioetins, especially interleukin-5 (IL-5), by an aberrant T-cell population [4]. Several common subsets of activated T lymphocytes were characterized, including CD3^−^ CD4^+^, CD3^+^ CD4^−^ CD8^−^, CD3^+^ CD4^+^ CD7^−^. L-HES patients usually present with a dermatological manifestation and should be closely monitored for progression to lymphoma [3].

Hypereosinophilic syndrome is a progressive and deceitful disorder often accompanied by a variety of complications. Cardiac involvement, usually endocardial fibrosis, could lead to fully symptomatic heart failure [10]. On the other hand, large-scale eosinophilic tissue infiltration and degranulation may result in massive venous and arterial thrombosis. Eosinophil peroxidase and major basic protein were found to promote platelet activation [11] and thrombus formation [12, 13]. In numerous studies, HES was found to be associated with different presentations of thrombosis [14–18], which, when uncontrolled, can lead to consumptive coagulopathy. Disseminated intravascular coagulation (DIC) is diagnosed based on clinical symptoms and laboratory parameters, including platelet count, prothrombin time, fibrinogen, and D-dimers, and could result in fatal bleeding complications [19]. The most commonly used medications used in HES are corticosteroids, hydroxyurea, interferon-*α*, tyrosine kinase inhibitors, immunosuppressants, and recently, anti-IL-5 antibodies, mepolizumab, and reslizumab [3, 4]. Corticosteroids are first-line therapy in most HES patients with a complete remission rate of over 80% within 1 month of treatment [20]. However, in some HES cases, the corticosteroid response may vary.

In this paper, we present a unique case of a patient with idiopathic hypereosinophilic syndrome who originally presented with acute abdominal pain and eosinophilic tumors within the colon wall found during laparotomy, complicated by a catastrophic thromboembolic event and disseminated intravascular coagulation. The patient signed an informed, written consent for the publication of his medical information and images.

## 2. Case Presentation

**Figure 1:**
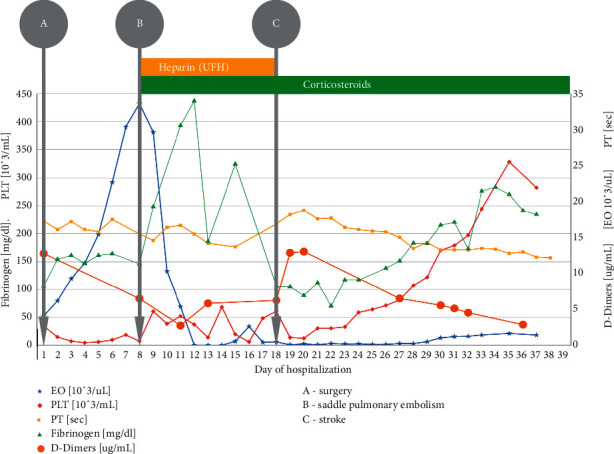
Timeline showing laboratory results changes, significant clinical events, and treatment.

**Figure 2:**
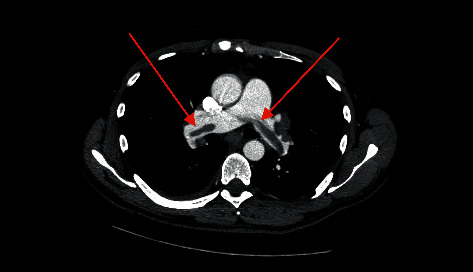
Chest angiotomography showing saddle pulmonary embolism.

The 35-year-old male was admitted to the emergency department due to persistent abdominal pain, fever, and emesis. Abdominal ultrasound revealed fluid in the right iliac fossa and widened intestinal loops. Laboratory results showed an elevated white blood cell count (WBC 19.43 × 10^3^/*μ*L), 10.5% eosinophils (AEC 2.05 × 10^3^/*μ*L), hemoglobin (Hgb) 14.8 g/dL, platelets (PLT) 232.8 g/dL, and CRP 300.6 mg/L. Initial coagulation tests were normal. The patient was admitted to the surgery department and on day one underwent exploratory laparotomy which revealed two tumors involving the ascending and transverse colon, as well as the greater curvature of the stomach (Figure 1). A biopsy was performed and the histopathological results demonstrated extensive eosinophilic infiltration. In the following days, the patient reported persistent abdominal pain and new extensive swelling and cyanosis of the lower extremities (bilateral phlegmasia cerulea dolens). Laboratory parameters changed significantly: WBC 52.72 × 10^3^/*μ*L, 63.77% eosinophils (AEC 33.62 × 10^3^/*μ*L), Hgb 10.5 g/dL, PLT 7.97 × 10^3^/*μ*L, D-dimers markedly elevated and fibrinogen level significantly decreased (146 mg/dl). Doppler ultrasound examination confirmed massive deep vein thrombosis and computed tomography angiography (CTA) showed portal vein thrombosis—unfractionated heparin was immediately introduced intravenously. Shortly, the patient developed hemodynamic and respiratory instability. Emergency chest CTA revealed saddle pulmonary embolism (Figure 2). The patient was transferred to the intensive unit of the cardiology department for treatment and monitoring. Supportive treatment along with corticosteroids and hydroxyurea for eosinophilia lowering therapy was used. Thrombocytopenia (10 × 103/*μ*L), elevated fibrin-related marker, e.g., D-dimer (13054 ug/L, ref. 0–278 ug/L), decreased fibrinogen level (70 mg/dl, ref. 200–472 mg/dl), and prolonged prothrombin time (16 sec., INR 1.46) were observed fulfilling the DIC criteria [19]. The patient required multiple packed red blood cells, platelet concentrate, and plasma transfusion during the overt DIC phase of the disease. A diagnostic workup of extreme eosinophilia was performed. The stool and serologic studies were negative for parasitic infections. The total IgE concentration was >2500 IU/ml (ref. 0–200 IU/ml). Genetic studies investigated the presence of FLIP1L1-PDGFRA translocation, CHIC2 deletion, JAK2 V617F mutation, and BCR-ABL1 fusion gene. All results were negative. Bone marrow aspirate cytometry did not show increased blast count or signs of clonal proliferation. Immunological tests showed the absence of ANCA (antineutrophil cytoplasmic antibodies), EMA (antiendomysial antibodies), and anti-DGP antibodies (antibodies against deamidated gliadin peptides). Therefore, there was no evidence of any ongoing autoimmune disease. The diagnosis of idiopathic HES was established.

**Figure 3:**
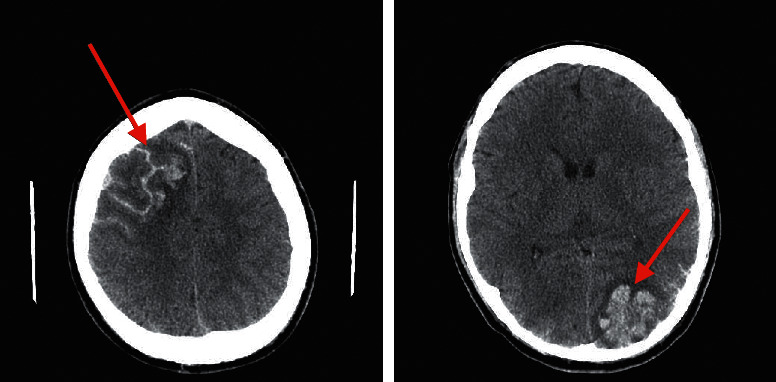
Head tomography showing cerebral infarction with secondary hemorrhagic transformation.

The clinical course of the patient was further complicated by headache and neurological symptoms: right amblyopia and left hemiplegia. A head CT scan was immediately performed and showed a cerebral infarct zone in the left occipital and right frontal lobes with secondary hemorrhagic transformation (Figure 3). TTE and TEE did not find signs of intracardiac thrombus. Protamine sulfate was administered to reverse the therapeutic effect of heparin. Fortunately, several days later, the neurological symptoms gradually improved and subcutaneous heparin was introduced. The eosinophil peripheral level was normalized after several days of corticosteroid therapy; however, the maintenance dose was prolonged. Laboratory signs of DIC resolved after 30 days of hospitalization. The prophylactic dose of low-molecular-weight heparin was prolonged. The patient was discharged on the 40th day of hospitalization in a good integrated condition. After 5 years of follow-up, the patient remains in good health with no hematological or thrombotic sequels.

## 3. Discussion

In this article, we report a case of idiopathic hypereosinophilic syndrome that led to DIC with tremendous thromboembolic complications. Physiologically, eosinophils with their cytoplasmic granules containing catalytic enzymes are responsible for nonspecific inflammatory and allergic reactions [21]. The gastrointestinal tract and pulmonary system are their primary residence areas, and the blood absolute eosinophil count only reflects tissue and organ concentration [21]. Eosinophilia can be of primary, clonal origin, or secondary to defined medical disorders. Eosinophilia is defined as an increased number of eosinophils over 600 cells per microliter of blood, while in the case of hypereosinophilia the value exceeds 1,500 cells [21]. Hypereosinophilia of unknown background accompanied by organ damage is an idiopathic hypereosinophilic syndrome [4]. Our patient initially presented moderate hypereosinophilia at a level of AEC 2.0 × 10^3^/*μ*L; however, during his clinical course, the eosinophil count increased significantly to more than 33.0 × 10^3^/*μ*L (over 66% of WBC). Data suggest that highly abundant eosinophils in the circulation provoke thrombosis [10–12, 22–24]. Wallace et al. in a retrospective analysis found that thrombosis affects about 20% HES patients, but rarely is it a presenting symptom [25]. The average AEC at the time of the thrombotic event was 15,000 × 10^3^/*μ*L. What is interesting is that in most cases analyzed, eosinophilia was untreated before thrombosis occurred. Probably, an early introduction of corticosteroids could effectively prevent life-threatening incidents.

HES-induced hypercoagulable state may eventually lead to overt DIC. However, scarce reports of such challenging cases are being published. In 1998, Yamada et al. described a young man who presented with abdominal pain, was diagnosed with HES and subsequently suffered a hemorrhagic stroke due to coagulation disturbances [26]. Uemura et al. reported a very interesting postmortem HES case of a man treated adequately with steroids but who suddenly died [27]. An autopsy revealed massive pulmonary emboli but also two solid eosinophilic tumors of the ascending and transverse colon walls. Our patient was primarily diagnosed with eosinophilic tumors at the same locations, and similarly, we observed massive pulmonary embolism with visible thrombus origin site in deep vein thrombosis of the lower extremities. Interestingly, we have also observed local vein thrombosis in the portal vein system, probably a direct effect of the presence of intra-abdominal mass. Uemura et al. have not reported signs of portal system vein thrombosis [27]. The authors suggested that large intestine tumors were responsible for the development of obstructive ileus, which would explain our patient's initial clinical presentation. Park et al. presented a case of idiopathic HES complicated by multiorgan infarctions and DIC [28]. This young female, similarly to our patient, suffered from two focal intracranial hemorrhages. The authors advocated for the continuation of anticoagulation therapy presuming thrombotic background of hemorrhage. We decided to temporarily stop anticoagulation. Surprisingly, both strategies produced favorable outcomes. In summary, we report a rare case of idiopathic hypereosinophilic syndrome complicated by thromboembolism and DIC, which was successfully treated with corticosteroids alone, and during a 5-year follow-up, the patient remains completely symptom-free.

## Data Availability

Data supporting this report are archived in general archive of University Hospital in Zielona Gora, Poland.
